# Behaviours and Beliefs Related to Whistleblowing Against Doping in Sport: A Cross-National Study

**DOI:** 10.3389/fpsyg.2022.835721

**Published:** 2022-05-03

**Authors:** Dmitriy Bondarev, Vassilis Barkoukis, Lambros Lazuras, Konstantin Bochaver, Despoina Oudra, Nikolaos Theodorou

**Affiliations:** ^1^Faculty of Sport and Health Sciences, University of Jyväskylä, Jyväskylä, Finland; ^2^Department of Physical Education and Sport Sciences, Aristotle University of Thessaloniki, Thessaloniki, Greece; ^3^Department of Psychology, Sociology and Politics, Sheffield Hallam University, Sheffield, United Kingdom; ^4^Laboratory of Sport Psychology, Moscow Institute of Psychoanalysis, Moscow, Russia; ^5^KEA Fair Play Hellas, Athens, Greece

**Keywords:** doping misconduct, reporting, trust, reasons for whistleblowing, thematic analysis

## Abstract

**Background:**

Whistleblowing has been recognized as an important deterrent of doping in elite competitive sport. The present study examined athletes’ knowledge of external whistleblowing channels and on how and where to report doping misconduct, perceived trust in different whistleblowing reporting channels, whistleblowing behaviour and athletes’ reasons for reporting (or not) doping misconduct.

**Methods:**

Athletes from Greece (*n* = 480), the Russian Federation (*n* = 512) and the United Kingdom (*n* = 171) completed a structured questionnaire on demographics, knowledge of different whistleblowing channels, perceived trust in internal and external whistleblowing channels, past whistleblowing behaviour and reasons for reporting (or not) doping misconduct.

**Results:**

The British athletes reported greater awareness of whistleblowing reporting channels (e.g., WADA’s Speak Up and IOC’s reporting platform) than did athletes from Greece (all *p* < 0.001) and Russia (*p* = 0.07, and *p* = 0.012) respectively. However, British athletes reported the lowest scores on knowledge of how and where to report doping misconduct, as compared to athletes from Greece and Russia. The majority of respondents reported greater trust to their coach or a club manager than to other whistleblowing channels, however, responses regarding other channels varied by country. Among athletes who detected doping misconduct 62% of athletes did not report it, while 38% reported it. Reasons for and against reporting doping misconduct reflected in eight themes that were identified using thematic analysis.

**Conclusion:**

Athletes showed low awareness of external whistleblowing channels and they predominantly trusted internal whistleblowing channels. Sportspersonship, confidence in resources and personal benefits were among the reasons that facilitate reporting doping misconduct. The present findings indicate that cultural context may play a role in the ways athletes perceive whistleblowing, and this should be taken into account by future interventions to promote the reporting of doping misconduct.

## Introduction

According to the World Anti-Doping Agency (2016), whistleblowing against doping misconduct refers to the disclosure of sensitive information about athletes and their entourage pertaining to Anti-Doping Rule Violations (ADRVs) as specified in the World Anti-Doping Code (World Anti-Doping Agency, 2016). Notable whistleblowing cases against doping misconduct in elite sport include Betsy Andreu who disclosed information about ADRVs in relation to Lance Armstrong’s doping in cycling, and Yuliya and Vitaliy Stepanovs who disclosed information about ADRVs in Russian athletics. Whistleblowing may occur at different levels, including reporting doping misconduct to relevant regional or national authorities, or to international whistleblowing reporting systems (WBRS), such as the World Anti-Doping Agency’s Speak Up platform, and the International Olympics’ Committee integrity and compliance hotline (Verschuuren, 2020). In January 2022, WADA’s President, Witold Bańka publicly praised whistleblowers and emphasised the importance of whistleblowing as a cornerstone of ADRV investigations:

“*[…]at the center of these investigations has been information soruced by whistleblowers or informants. These are the unseen heroes of anti-doping […] The athlete community and the global anti-doping system are extremely grateful for their efforts. They are making a real difference for the integrity of sport.*”

In an additional effort to promote whistleblowing and encourage athletes to speak up against doping misconduct, the revised 2021 World Anti-doping Code included provision to protect whistleblowers by defining an ADRV as any act or attempt of third parties to hinder the whistleblowing process or to retaliate against whistleblowers. Although at an institutional level WADA and other sport governing bodies actively promote whistleblowing, still at a behavioural level, athletes and their entourage, and sport stakeholders may not yet fully endorse whistleblowing as a way to tackle doping misconduct. Qualitative research in this area has indicated that although the majority of student athletes from the United States and the United Kingdom perceived doping as serious misconduct, still more than half of them were reluctant to reporting it, and even if they decided to report it, only a small minority would do so *via* official WBRS (Erickson et al., 2017). Accordingly, a qualitative study with athletes, coaches, and sport governing body directors, indicated that although the value of whistleblowing as a deterrent of doping was clear and whistleblowing was largely endorsed, still social and relationship dynamics, as well as concerns over fears of retaliation and anonymity of the whistleblowing report presented barriers to whistleblowing (Barkoukis et al., 2021b). Further research on whistleblowing against doping used quantitative research methods to explore how individual differences in athletes’ motivation and moral orientations were associated with whistleblowing intentions. It was demonstrated that athletes with higher levels of autonomous motivation and moral functioning, as indicated in higher scores in sportspersonship orientations (e.g., following rules, respecting officials and opponent, engaging in pro-social behaviour in sport), were also more likely to want to report witnessed or suspected doping misconduct through WBRS (Barkoukis et al., 2021a). Another recent study further indicated that considerations of social norms (e.g., if whistleblowing is socially approved by referent others, and if most similar others would report doping misconduct *via* WBRS) were also associated with intention to engage in whistleblowing among elite competitive athletes from three countries (Lazuras et al., 2021). This study also indicated that, on average, athletes displayed relatively strong intention to report doping misconduct *via* WRBS.

Taken together, the extant (but limited) research on whistleblowing against doping misconduct indicates that further investigation into athletes’ beliefs, social norms, and dispositions with regards to whistleblowing can be helpful in understanding how the decision to report doping misconduct is shaped. To this end, lessons we can learn from other settings (e.g., in corporate, financial, and/or public administration organizations) where whistleblowing research is more extended, can potentially elucidate the range of factors that may be relevant to the athletes’ decision to report doping misconduct *via* official WBRS (see also Vandekerckhove and Phillips, 2019; Verschuuren, 2020).

Different reviews of whistleblowing studies in corporate and public administration organisations have shown that there is a distinction between using internal (in-house) and external WBRS (Loyens and Vandekerckhove, 2018; Park et al., 2020). Employees who tend to resort to external WBRS are more likely to possess more conclusive evidence about the wrongdoing in question, have higher power or status within the organization, are more concerned about ethical (vs. corporate) values, and less concerned with the damage their reporting may inflict on their organization (Mesmer-Magnus and Viswesvaran, 2005; Culiberg and Mihelič, 2017; Lee and Xiao, 2018). Furthermore, the whistleblowers’ demographic characteristics, such as age, sex, and education can be associated with whistleblowing intentions and actual behaviour. Specifically, although the findings about age and sex effects are mixed, females and older employees (who possibly assume higher tenure and, consequently, higher-power positions within an organization) appear to be more likely to blow the whistle (Culiberg and Mihelič, 2017; Gao and Brink, 2017). Empirical research has also indicated that perceived trust (i.e., trust in the reporting authority, and trust in the reporting system itself) is strongly associated with willingness to report misconduct, mainly because perceived trust can alleviate the perceived risks of whistleblowing (Lowry et al., 2013). Individuals are more likely to want to report wrongdoing if they perceive the authority they report to, and the reporting channel/system they use, as trustworthy, and if they have a sufficient level of knowledge of what to report, how, and what reporting options are available (Berry, 2004; Lowry et al., 2013).

These findings have important implications for whistleblowing research in the context of doping misconduct. First of all, there is a dearth of doping-related research examining individual-level characteristics of whistleblowers, such as age and sex. Secondly, the extant studies on whistleblowing against doping have not systematically addressed the role of trust and subjective knowledge of whistleblowing procedures. Although WADA has invested significant resources into the development of the Speak Up platform, this WBRS represents an external reporting channel, and it is not yet documented how many athletes are aware of this WBRS, and whether they trust this more than other reporting channels or systems (e.g., speaking to a coach).

It is also important to identify the reasons for which athletes may (or may not) decide to report doping misconduct. According to Behavioural Reasoning Theory (Westaby, 2005) investigating people’s reasons for their decision can help in better understanding the motivational impetus that underlies given behaviours. Therefore, investigating the reasons why athletes decide to report doping misconduct, or stay silent, can help in modelling their behaviour more effectively, as those reasons reflect how attitudes, social norms, and self-efficacy beliefs are formed. Reasons can also shape intentions directly, thereby having a more direct impact on the decision-making process underlying whistleblowing (Westaby et al., 2010).

The present study is an exploratory one and aimed to further extend the existing literature in whistleblowing against doping misconduct by emphasizing the roles of: (a) demographic characteristics of athletes (i.e., age and sex); (b) knowledge of external and online WBRS, such as WADA’s Speak Up platform, and IOC’s Integrity and Compliance hotline; (c) subjective knowledge about how and where to report doping misconduct; (d) perceived trust in different WBRS, ranging from using WADA’s online reporting platform (external whistleblowing), to talking to a coach (internal whistleblowing); and (e) athletes’ reasons for reporting (or not) doping misconduct, should they become aware of it.

## Materials and Methods

### Sample and Recruitment

The data reported in the present article was drawn from a larger-scale international study that investigated the psychosocial factors associated with whistleblowing against doping misconduct among competitive athletes, and the sampling methodology has been reported in detail elsewhere (Lazuras et al., 2021; Barkoukis et al., 2021a). Overall, 1,163 athletes from both individual and team sports from Greece, the Russian Federation, and the United Kingdom were recruited and their details are as follows: 480 competitive athletes (283 males, *M*_age_ = 19.88, SD = 1.70) from Greece; 512 competitive athletes (341 males, *M*_age_ = 20.08, SD = 5.49) from the Russian Federation; and 171 competitive athletes (121 males, *M*_age_ = 20.31, SD = 1.95) from the United Kingdom.

Participants were informed about the aims and purposes of the study, as well as their participation rights either face-to-face (Greece) or online (Russian Federation and United Kingdom). Participants completed the questionnaires anonymously, after providing their informed consent. The instructions regarding the completion of the questionnaire were similar in all countries.

#### Ethical Approval

The study was conducted in line with the guidelines of the British Psychological Society’s Code of Human Research Ethics, and the study obtained ethical approval from the respective boards of the participating institutions.

### Survey

The measures reported in this manuscript included demographic characteristics (age and sex); awareness of online external WBRS (i.e., WADA’s Speak Up platform and IOC’s Integrity and Compliance hotline); subjective knowledge about reporting doping misconduct; perceived trust in different internal and external whistleblowing channels and systems; past whistleblowing behaviour; and behavioural reasons for deciding to report (or not) doping misconduct.

To ensure that all participants had the same understanding of whistleblowing the following definition was used: “*Whistleblowing is defined as the disclosure of sensitive information about athletes and/or their entourage (e.g., coaches, managers, and trainers) with respect to any suspected: (a) Anti-Doping Rule Violation, (b) World Anti-Doping Code (Code) non-compliance violation, and (c) Act or omission that could undermine the fight against doping.*” This definition is consistent with the description of whistleblowing against doping as presented in WADA’s Whistleblowing Program (World Anti-Doping Agency, 2016).

#### Demographics

Participants reported their *age* (in years) and *sex* (male, female, or other).

*Knowledge of external and online WBRS* was assessed by asking participants if they were familiar WADA’s Speak Up platform, and with IOC’s Integrity and Compliance hotline, and binary (yes/no) responses were recorded.

#### Subjective Knowledge About How and Where to Report Doping Misconduct

Participants were presented with the stem proposition “*If I had detected, identified, witnessed or knew of, or had reasonable grounds for suspecting that doping misconduct had occurred*,” followed by two different responses capturing subjective knowledge: *“I would know exactly how to report it,”* and *“I would know exactly where to report it.”* Responses were recorded on a 7-point scale ranging from 1 (=strongly disagree) to 7 (=strongly agree). A mean score was computed and with higher scores reflected greater subjective knowledge over whistleblowing.

*Perceived trust in internal and external whistleblowing channels* was measured with a single question: *“If you wanted to report doping misconduct, how much would you trust each of the following sources?*,” followed by seven response options reflecting external reporting channels/systems (i.e., WADA’s Speak Up platform; IOC’s integrity and compliance hotline; NADO (a National Anti-Doping Organization); an anonymous whistleblowing platform that is independent from sport; police) and internal reporting channels (i.e., my coach; my club/team manager). Responses were recorded on a 5-point continuous scale, ranging from 1 (=not at all) to 5 (=very much). A mean score was computed and with higher scores indicated greater trust.

*Past whistleblowing behaviour* was measured by asking participants to indicate if they ever detected, identified, witnessed or knew of, or had reasonable grounds for suspecting that a doping misconduct occurred. Three response options were presented: (1) = No, I never detected, identified, witnessed or knew of, or had reasonable grounds for suspecting that a doping misconduct occurred; (2) = Yes, I detected, identified, witnessed or knew of, or had reasonable grounds for suspecting that a doping misconduct occurred, and I decided not to report it; (3) = Yes, I detected, identified, witnessed or knew of, or had reasonable grounds for suspecting that a doping misconduct occurred, and I reported it.

*Athletes’ reasons for reporting (or not) doping misconduct* were recorded using the elicitation method. Instead of providing a pre-determined set of reasons, the elicitation method allows participants to reflect on their own personal reasons for deciding to act (or not to act), and this method has been recommended by the proponents of behavioural reasoning theory (Westaby, 2005; Westaby et al., 2010). Because reasons can be context-specific and subjective, and whistleblowing behaviour against doping misconduct is a rather understudied topic and relevant quantitative measures are lacking, we deemed appropriate to utilise the elicitation method to identify the most commonly referred reasons for engaging in (or abstaining from) whistleblowing and, therefore, participants were presented with a relevant open-ended question asking them to report the main reasons they decided to report (or not) doping misconduct.

### Data Analysis

Descriptive analysis of the measurements responses using mean, standard deviations (SDs) and 95% confidence intervals (CIs) for quantitative variables or frequencies and proportions for categorical variables was performed. The differences between countries in the studied quantitative variables were tested by analysis of variance (ANOVA) with the *post-hoc* Tukey test. For categorical variables, differences were tested with a chi-square test in R package fifer (Fife, 2017). A two tailed *p*-value of less than 0.05 was accepted as significant.

Data related to the reasons for reporting or not reporting doping misconduct were analysed using thematic analysis to categorise the respondents’ reasons by themes (Aronson, 1995). Themes were constructed by the authors based on the participants’ responses. Through the iterative analysis of each response, themes emerged, and the authors then aggregated similar responses by themes. If discrepancies on a response categorisation between authors were arise it was resolved by discussion and consensus. Number of responses as well as examples of responses for each theme are given and they were edited to correct spelling, omit extraneous words or to protect identities.

## Results

### Demographics

The mean age of the participants and a distribution by sex were similar across countries (Table 1).

**Table 1:** Participants’ descriptive characteristics, awareness of whistleblowing channels and knowledge on reporting doping misconduct across countries.

	Greece	Russia	UK	p^*^
*Demographic characteristics*				
Age, years	*n* = 457 19.8 (1.7)	*n* = 498 20.1 (5.4)	*n* = 167 20.3 (1.9)	0.457
Sex (male), *n* (%)	*n* = 456 283 (67)	*n* = 512 341 (67)	*n* = 170 121 (71)	0.588
*Knowledge of external and online WBRS*				
WADA’s Speak Up!, (yes), *n* (%)	*n* = 444 68 (15)	*n* = 504 162 (32)	*n* = 39 18 (46)	**<0.001** Gr-Ru <0.001 Gr-UK <0.001 Ru-UK 0.07
IOC’s Integrity and Compliance hotline, (yes), *n* (%)	*n* = 420 28 (7)	*n* = 497 125 (25)	*n* = 67 27 (40)	**<0.001** Gr-Ru <0.001 Gr-UK <0.001 Ru-UK 0.012
*Subjective knowledge about how and where to report doping misconduct*				
Knowledge how to report, mean (95% CI)	*n* = 471 3.64 (3.47; 3.81)	*n* = 511 4.40 (4.24; 4.56)	*n* = 157 3.21 (2.90; 3.51)	**<0.001** Gr-Ru <0.001 Gr-UK 0.033 Ru-UK <0.001
Knowledge where to report, mean (95% CI)	*n* = 471 3.62 (3.43; 3.81)	*n* = 511 4.28 (4.10; 4.45)	*n* = 158 3.21 (2.89; 3.51)	**<0.001** Gr-Ru <0.001 Gr-UK 0.073 Ru-UK <0.001

Gr, Greece; Ru, Russia; UK, the United Kingdom.

* *t*-test for continuous variables or Chi-square test in the case of categorical variables. Bold values indicate statistically significant results.

### Knowledge of External and Online WBRS and Knowledge About How and Where to Report Doping Misconduct

In total, 248 athletes (21%) reported that they aware of the WADA’s Speak Up platform and 180 athletes (15%) aware of the IOC’s Integrity and Compliance hotline (Table 1). Further, significant differences across countries in the knowledge of WADA’s Speak Up platform [*F*(2, 984) = 23.5, *p* < 0.001] and IOC’s Integrity and Compliance hotline [*F*(2, 981) = 42.0, *p* < 0.001] were observed. The *post-hoc* analysis revealed that Greek athletes were significantly less aware of the official international WBRS, such as WADA’s Speak Up and IOC’s Integrity and Compliance hotline, as compared to Russian and British athletes. Similarly, significant differences across countries in the knowledge of how to report [*F*(2, 1,135) = 32.9, *p* < 0.001] and where to report [*F*(2, 1,137) = 21.8, *p* < 0.001] doping misconduct were detected. Specifically, the Russian athletes reported significantly greater scores on knowledge of how and where to report doping misconduct, than Greek and British athletes (Table 1).

### Perceived Trust in Internal and External Whistleblowing Channels

Analyses of the degree of trust in reporting doping misconduct to different actors have shown that the athletes would predominantly trust their coach or a club manager (Table 2). This was evident from greater scores for degree of trust in the coach and comparison of respective confidence intervals. However, the degree of trust in other actors varied between countries. Specifically, the Greek athletes would trust more the WADA’s platform or police than their national anti-doping organisation. The Russian athletes, in contrast, would trust police to a lesser extent than RUSADA and the official platforms run by WADA and IOC. The British athletes would trust more UKAD as well as the WADA and IOC platforms to disclose doping misconduct, but less so the police or an anonymous platform.

**Table 2:** Trust in different whistleblowing reporting systems.

Whistleblowing reporting systems	Greece	Russia	UK	p^*^
WADA’s Speak Up! Platform, mean (95% CI)	*n* = 467 2.92 (2.79; 3.05)	*n* = 511 3.47 (3.38; 3.57)	*n* = 132 3.19 (2.95; 3.43)	<0.001 Gr-Ru <0.001 Gr-UK 0.076 Ru-UK 0.049
IOC’s Integrity and Compliance hotline, mean (95% CI)	*n* = 467 2.58 (2.46; 2.70)	*n* = 511 3.41 (3.31; 3.50)	*n* = 131 3.09 (2.87; 3.34)	<0.001 Gr-Ru <0.001 Gr-UK <0.001 Ru-UK 0.020
National platforms (respectively ESKAN /RUSADA/UKAD), mean (95% CI)	*n* = 464 2.42 (2.20; 2.53)	*n* = 511 3.35 (3.24; 3.46)	*n* = 132 3.61 (3.40; 3.85)	<0.001 Gr-Ru <0.001 Gr-UK <0.001 Ru-UK 0.005
An anonymous independent online platform, mean (95% CI)	*n* = 466 2.86 (2.72; 3.00)	*n* = 511 2.75 (2.64; 2.85)	*n* = 130 2.50 (2.26; 2.73)	0.032 Gr-Ru 0.479 Gr-UK 0.025 Ru-UK 0.144
Police, mean (95% CI)	*n* = 471 2.93 (2.81; 3.04)	*n* = 511 2.57 (2.46; 2.68)	*n* = 131 2.59 (2.35; 2.81)	<0.001 Gr-Ru <0.001 Gr-UK 0.033 Ru-UK 0.905
Respondents’ coach, mean (95% CI)	*n* = 472 2.94 (2.79; 3.07)	*n* = 511 4.00 (3.90; 4.09)	*n* = 132 3.61 (3.40; 3.83)	<0.001 Gr-Ru <0.001 Gr-UK <0.001 Ru-UK 0.020
Respondents’ club/team manager, mean (95% CI)	*n* = 471 2.55 (2.45; 2.68)	*n* = 511 3.51 (3.41; 3.62)	*n* = 131 3.45 (3.21; 3.45)	<0.001 Gr-Ru <0.001 Gr-UK <0.001 Ru-UK 0.089

Gr, Greece; Ru, Russia; UK, the United Kingdom.

* ANOVA post-hoc values.

### Past Whistleblowing Behaviour

Nearly one-fifth (*n* = 237, 21%) of the 1,163 surveyed participants gave their reasons for reporting (or not) doping misconduct (Table 3). Of those 237 athletes, 148 athletes (62%) that they decided not to report doping misconduct, and 89 (38%) athletes responded. In comparison with the Greek sample, significantly more Russian and United Kingdom athletes had detected, identified, witnessed or knew of, or had reasonable grounds for suspecting that doping misconduct occurred, and reported it. On the other hand, the proportion of athletes who had detected, identified, witnessed or knew of, or had reasonable grounds for suspecting that doping misconduct and did not report it, was significantly higher in the Greek sample in comparison with the Russian or United Kingdom athletes (Table 3).

**Table 3:** Whistleblowing behaviour.

	Greece *n* = 480	Russia *n* = 512	UK *n* = 171	p^*^
Never detected doping misconduct, *n* (%)	375 (78)	393 (77)	114 (86)	**<0.001** Gr-Ru <0.001 Gr-UK <0.001 Ru-UK 0.075
Detected doping misconduct but did not report it, *n* (%)	93 (20)	46 (9)	9 (7)	
Detected doping misconduct and reported it, *n* (%)	7 (2)	72 (14)	10 (7)	

Gr, Greece; Ru, Russia; UK, the United Kingdom.

* Chi-square test. Bold values indicate statistically significant results.

### Athletes’ Reasons for Reporting (or Not) Doping Misconduct

Of those 237 responses that provided reasons for reporting or not reporting doping misconduct, 69 responses were either partially completed or not completed answers, and therefore, they were discarded from subsequent thematic analyses. Given the low number of responses in the reported reasons across countries, the thematic analysis was conducted for the whole sample (Table 4). The authors analysed the 168 responses and identified, respectively, five themes from the responses of those who decided not to report doping misconduct, and three themes emerged from the responses of those who decided to report doping misconduct.

**Table 4:** Athletes’ reasons for reporting (or not) doping misconduct: emerged themes and responses collected from the open-ended question.

Themes	*n* (%)	Example of responses
*Yes, I identified doping misconduct and decided not to report it, n = 116*		
It’s not my problem	43 (37)	It is not my business. It did not affect me personally. It is pointless. I think it would not help. It does not depend on me. It did not concern me.
Fear of consequences	32 (27)	I did not want to get in trouble. Consequences for my career. Revenge from colleagues. Danger. Bullying.
Team “code of silence”	19 (17)	He was a teammate. I am not that kind of man. It would be bad for a team. Solidarity.
Lack of knowledge or trust	12 (10)	I do not know how to report. Whom to report to? I did not have the way to do so. Do not trust the source that I know.
Reluctance due to the lack of evidence	10 (9)	I did not have enough proof (that other used banned substances). I must be sure for 100%. I only heard of such cases.
*Yes, I identified doping misconduct and reported it, n = 52*		
Sportspersonship	33 (63)	Sport is for fair competition. To uphold honour in sport, It is [using banned substances] against the spirit of sport.
Confidence in resources	14 (27)	I think that it is necessary to report. There is a hotline here, and I was confident to use it. It is an anonymous channel. I can preserve my privacy.
Personal benefits	5 (10)	I want to receive some stimulation [incentives]. This [doping misconduct] was too obvious, and I wanted to feel good with myself.

The emerged themes, final number of responses within a theme and a theme proportion with illustrative examples of responses are shown in the Table 4. Among athletes who decided not to report doping misconduct, the theme “It’s not my problem” accounted for the greater proportion of responses, following by such themes as “Fear of consequences,” “Team code of silence,” “Lack of knowledge or trust” and “Reluctance due to lack of evidence.” Among athletes who decided to report doping misconduct the greater proportion was accounted by the theme “Sportspersonship” following by themes “Confidence in resources” and “Personal benefits.”

The first theme “It is not my problem” reflects reasons that relate either to indifference about other athletes’ doping and violations of anti-doping rules, or reduced self-efficacy or ability to influence the course of such actions (e.g., reduced efficacy to prevent others from doping). These meanings are evidenced by the following quotes:

“*It [reporting doping misconduct] is not my business*”, “*It did not affect me personally*”, “*It is pointless*”, “*I think it would not help*”.

The second theme “Fear of consequences” represents responses related to expression of consequences for athletic career or fear of retribution from peers. These meanings are evidenced by the following quotes: “*I did not want to get in trouble*,” “*Consequences for my career*”, “*Revenge from colleagues*”, “*Bullying*,” “*Fear*”, “*Afraid of a revenge*,” and “*I thought it was dangerous*.”

The third theme “Team code of silence” represents responses related to expression of adherence to norms of loyalty and staying quite to protect a reputation of peers. These meanings are evidenced by the following quotes: “*He was a teammate*”, “*I am not that kind of man [who speak up against peers]*,” “*It [reporting a doing misconduct] would be bad for a team*,” “*Solidarity*,” “*We are a family*.”

The fourth theme “Lack of knowledge or trust” represents responses related to expression not knowing how and where to report doping misconduct, or lack of trust to a channel for reporting it. These meanings are evidenced by the following quotes: “*I did not know how to report it [doping misconduct]*,” “*Whom to report to?*,” “*I did not know what I should do*,” “*Do not trust the source that I know*.”

The fifth theme “Reluctance due to lack of evidence” represents responses related to lack of clear evidence to be sure in occurrence of doping misconduct. These meanings are evidenced by the following quotes: “*I did not have enough proof [that other used banned substances]*,” “*I must be sure for 100%*,” and “*I only heard of such cases [from someone else]*.”

The following three themes represent responses of those who identified doping misconduct and reported it. The theme “Sportspersonship” reflects the desire of fair competition and protection of sport integrity. This was evidenced by the following quotes: “Sport is for fair competition,” “To uphold honour in sport,” It is [using banned substances] against the spirit of sport,” “Sport should be clean,” “Culture should be better.”

The theme “Confidence in resources” represents responses related to expression of trust in the resource for reporting doping misconduct or self-confidence in own actions. These are reflected in the following quotes: “*I think that it is necessary to report*,” “*There is a hotline here, and I was confident to use it*,” “*It is an anonymous channel*,” “*I can preserve my privacy*.”

The theme “Personal benefits” represents responses related to expression of getting personal benefits from reporting doping misconduct, and was reflected in the following quotes: “*I want to receive some stimulation [incentives]*”, “*This [doping misconduct] was too obvious, and I wanted to feel good with myself*.”

## Discussion

This study has examined beliefs and behaviours in relation to reporting doping misconducts across three samples of competitive athletes from Greece, the Russian Federation and the United Kingdom. The present study is the first one conducting with the international participants and reporting on knowledge of external and online WBRS for doping misconduct, subjective knowledge about how and where to report doping misconduct, perceived trust in different WBRS, and whistleblowing behaviour and athletes’ reasons for reporting (or not) doping misconduct.

### Knowledge of External WBRS

This study showed that the vast majority of competitive athletes were not aware of either the WADA’s Speak Up platform (75%) or the IOC’s Integrity and compliance platform (81%). Between country analyses further showed that this was mostly the case among Russian and Greek athletes, than British ones. One of the possible explanations is that the WADA and IOC platforms are available in English (but nor in Russian or Greek), therefore, native English speakers may be more aware of them. One way to address the language barrier is to offer international reporting channels, such as WADA’s Speak Up and IOC’s hotline in other languages.

### Subjective Knowledge About How and Where to Report Doping Misconduct

Furthermore, we observed that significant cross-country differences emerged in subjective knowledge about how and where to report doping, with Russian athletes reporting greater knowledge levels than British and Greek athletes. One way to improve athletes’ knowledge of how and where to report doping misconduct is by raising awareness through related education initiatives and campaigns. Potentially, such opportunities may include a coach or club officials. As an example, in 2020 United Kingdom Anti-Doping developed the “Protect Your Sport” campaign^1^ to raise awareness about whistleblowing and ways to provide anonymous and confidential reports of ADRVs.

### Perceived Trust in Different WBRS

In all countries, the majority of the athletes had reported that they trust predominantly internal reporting channel – their coach or club manager to disclose doping misconduct, while lower trust was noticed for external channels (e.g., an anonymous platform, WADA or IOC platforms). As significant resources has been invested in developing whistleblowing platforms, external reporting channels seem not attract a high number of informants (Erickson et al., 2019). Literature from public sectors showed that external channels are preferred when a whistleblower has less concerns about harming the organizational reputation (Chen and Lai, 2014). It has been shown that sport is characterized by intense loyalty and organizational silence compared to public sectors (Babiak and Wolfe, 2009). Our finding may suggest that the coach or other team personnel may be specific agents that influence whistleblowing behaviour in athletes. This finding corroborates Erickson et al. (2017) who showed that athletes, instead of directly reporting doping misconduct, they would prefer to discuss this issue with a violator or to report this to a coach, so that a coach would take necessary action. Thus, it could be useful to deliver and convey coach-targeted (or other athlete-supporting personnel) educational messages related to whistleblowing issues. One such a message could be developed around a positive culture and norms towards whistleblowing. Such a culture and norms may instil favourable attitudes towards whistleblowing and thus, whistleblowing intention (Park and Blenkinsopp, 2009). It has been shown that agents with supervisory status are more likely to blow the whistle (Verschuuren, 2020). Thus, another possible avenue for involving coaches in promoting whistleblowing is their perceived supervisory status and trustworthiness.

Furthermore, with respect to trust to different WBRS we also observed some variability across countries. The Greek athletes would trust their NADO to a lesser extent than WADA or other anonymous platforms, while the Russian and British athletes would trust more to their NADO as WADA or IOC whistleblowing platforms than to anonymous platform or police. There are two plausible explanations for these findings, one relating to cross-cultural differences in clean sport, and the other reflecting institutional infrastructure to support whistleblowing locally. Recently, it has been shown that athletes’ perceptions of the importance of clean sport and clean sport values can vary across different countries (Woolway et al., 2021). As values are linked to doping behaviour (Ring et al., 2020), it could be that culture may also play a role in engagement with, and acceptance of whistleblowing in sport (Keenan, 2007; Bondarev et al., 2021). Culture refers to the individual’s characteristics that based on the perception of rules, norms, roles, and values, influenced by various societal levels (e.g., country, race, occupation etc.; Triandis, 1972). It has also been shown that cultural characteristics (a country level) play a role in promoting active support for anti-doping policies (Barkoukis et al., 2022). If this is the case, then our finding suggests that whistleblowing education and awareness-raising campaigns should consider country-specific values around clean sport. Another plausible explanation with regards to our findings regarding the Greek athletes’ trust to NADOs is that, until recently, Greece lacked resources for reporting doping misconduct. The newly re-constituted NADO in Greece^2^ currently includes an intelligence and investigations section, but this was not the case when the data collection for the present study took place.

### Whistleblowing Behaviour and Athletes’ Reasons for Reporting (or Not) Doping Misconduct

The limited literature on whistleblowing behaviour in doping context gives a scarce understanding of its prevalence. In this study, we observed that there is a country specific difference in doping whistleblowing behaviour. While across all countries most of the athletes reported that they themselves had never detected doping misconduct, the proportion of athletes differs in terms of those who detected doping misconduct and reported it and those who did not report it. Specifically, the Russian sample consists of higher proportion of those who detected but did not report doping misconduct. While the Greek sample had more athletes, who reported doping misconduct.

Blowing the whistle on doping may represent diverse forms of the behaviour and not only reflect reporting or non-reporting to authorities. Publicly available incidents of doping whistleblowing in sports are few; however, our results indicated that among the whole sample every fifth participant had encountered a choice related to whistleblowing.

The present study identified some of the reasons why athletes may report (or not) doping misconduct. Specifically, the major reason for not reporting doping misconduct was a low ethical commitment. Athletes’ responses exemplified that whistleblowing is not their business or they perceive it as pointless. The fear of consequences was the second largest category among the reasons not to report doping misconduct. Potential whistleblowers may experience a number of consequences for this behaviour, such as fear of retribution, discrimination or rejection by others (Kirby et al., 2011).

Athletes may be hesitant to blow the whistle against doping also due to confronting with the moral dilemma such as report a doping to protect clean sport and not to report a doping to preserve a reputation of their sport. Such a dilemma was exemplified in our study as the emerged theme related to “code of silence” when athletes indicated on solidarity with a peer who use doping. This may highlight the role of social norms and expectations in whistleblowing behaviour (Whitaker et al., 2014). Lack of knowledge on how to report and trust to the reporting channel was also mentioned by the respondents who preferred not to blow the whistle against doping. Interestingly, participants indicated that reluctance to report doping misconduct may be due to the lack of evidence for use of banned substance. From the organizational point of view, the above examples may reflect the need of better introduction of communication channels within the organization as well as outside of an organization (e.g., independent organization) to promote whistleblowing against doping.

The following themes were emerged among reasons for reporting doping misconduct: sportspersonship, confidence in resources and personal benefits. Sportspersonship has been shown to associate with moral behaviour in sport, rule compliance and prosocial behaviour (Chantal et al., 2005). Our results indicate that sportspersonship may be positively related to whistleblowing behaviour. Confidence in resources that reflect knowing a trustful recourse to report doping misconduct may encourage athletes to blow the whistle against doping. Providing a confidential source for reporting could help to reduce the perceived risk for potential whistleblowers (Gundlach et al., 2003). Interestingly, among reasons that promote reporting of doping misconduct there were also the desire to receive incentives for this behaviour. Further studies are needed to investigate to what extend doping whistleblowing may be related to maladaptive behaviour or be a product of ego orientation. Possibly, to divert such a motivation from maladaptive (e.g., money incentives or selfish motives) to more adaptive source a clear message that efforts of being proactive in protecting clean sport are well encouraged by a sporting community is needed.

## Study Limitations

Although, this is the first cross-country study reporting on the beliefs and behaviour related to whistleblowing against doping, there are some limitations worth mentioning. First, whistleblowing behaviour was self-reported. Self-report measures are subject to social desirability bias and may lead to overestimation of the prevalence of reporting doping misconduct. Athletes were not asked to provide evidence for the reporting doping misconduct so that to eliminate a possibility of linking this information with an individual and to preserve anonymity. Second, number of participants from the United Kingdom were considerably lower than from Greece and Russian Federation, which may influence statistical power when assessing differences across countries. Even though, the reported differences points to the importance of conducting country-specific research on beliefs regarding whistleblowing against doping among athletes, in terms of whistleblowing behaviour our analysis was performed on the unified sample. Thus, identified themes of reasons of whistleblowing behaviour represents international athletes’ viewpoint.

On the other hand, reasons for reporting (or not) doping misconduct may partially be dependent on a perception about a seriousness of wrongdoing (Ayers and Kaplan, 2005; Vandekerckhove and Phillips, 2019). Such a perception in turn could rely on knowledge related to doping or performance enhancement substances and vary across countries (Barkoukis et al., 2022).

Although the present study was exploratory and did not address culture-specific variables, future research may examine how cultural characteristics, such as values, relate to whistleblowing behaviour.

Despite those limitations, the present study has important practical implications for policy-makers and antidoping practitioners. Our findings suggest that promoting whistleblowing behaviour may require understanding of cultural factors facilitating whistleblowing. This is a crucial issue for international enterprise such as sport which covers very diverse local and organisational context. In addition, identified reasons for which athletes may (or may not) decide to report doping misconduct can help better understand the motivational forces that drives whistleblowing behaviour (Westaby, 2005). Our results suggests that measures facilitating whistleblowing could benefit from adapting a more holistic approach to existing that takes into account not just the characteristics of the whistleblowers but also the characteristics of the wrongdoing and the wrongdoer (coach, club manager, high profile athlete), as well as the characteristics of the WBRS/report recipient (Gao and Brink, 2017). On the basis of the present findings, we recommend that policy and/or education initiatives to promote whistleblowing against doping in sport should consider highlighting the benefits of whistleblowing; help athletes, coaches, and other athlete support personnel who may serve as whistleblower to resolve ethical or other dilemmas (e.g., decisional imbalance); and explicitly offer the necessary provisions to protect whistleblowers, including legal, physical, or psychological support.

## Conclusion

This study is the first that reports on beliefs and behaviours in relation to reporting doping misconducts across international samples of competitive athletes. The results showed that athletes had low awareness of external whistleblowing channels. They predominantly trusted internal whistleblowing channels, while trust to external whistleblowing channels was clearly weaker. Promoting whistleblowing against doping in sport would potentially benefit by taking into account the identified themes underlying reasons for whistleblowing behaviour.

## Data Availability Statement

The raw data supporting the conclusions of this article will be made available by the authors, without undue reservation.

## Ethics Statement

The studies involving human participants were reviewed and approved by Sheffield Hallam University. Written informed consent for participation was not required for this study in accordance with the national legislation and the institutional requirements.

## Author Contributions

DB, the corresponding author, compiled, analysed and interpreted the data and drafted the original manuscript. VB and LL obtained funding, designed and supervised the entire study. DB, KB, DO, NT were responsible for data collection. All authors contributed to the article and approved the submitted version.

## Funding

This work was supported by the Social Science Research Grant program of the World Anti-Doping Agency in 2017.

## Conflict of Interest

The authors declare that the research was conducted in the absence of any commercial or financial relationships that could be construed as a potential conflict of interest.

## Publisher’s Note

All claims expressed in this article are solely those of the authors and do not necessarily represent those of their affiliated organizations, or those of the publisher, the editors and the reviewers. Any product that may be evaluated in this article, or claim that may be made by its manufacturer, is not guaranteed or endorsed by the publisher.

## Footnotes

^1^ https://www.ukad.org.uk/protect-your-sport

^2^ www.eokan.gr
